# Analysis of different plant- and animal-based dietary patterns and their relationship with serum uric acid levels in Chinese adults

**DOI:** 10.1186/s12937-023-00885-2

**Published:** 2023-10-27

**Authors:** Danhui Mao, Jin Feng, Yangzilin Zhou, Honggang Li

**Affiliations:** Health Management and Policy Research Center, School of Management, Shanxi Medical University, 030000 Taiyuan, China

**Keywords:** Plant-based diet, Animal-based diet, Dietary pattern, Serum uric acid, Hyperuricemia

## Abstract

**Background:**

Dietary patterns play an important role in regulating serum uric acid levels in the body, but evidence for the association between different kinds of plant-based and animal-based dietary patterns and individual serum uric acid levels is scarce and inconsistent.

**Methods:**

We analyzed data from the sixth wave of the China Health and Nutrition Survey. The plant-based diet of 7,806 participants was determined using three consecutive 24-hour dietary recalls, and latent profile analysis was used to identify dietary patterns among participants. Serum uric acid levels were analyzed using the enzymatic colorimetric method. The association between intakes of different types of dietary pattern and individual serum uric acid levels was analyzed using linear regression analysis, after adjusting for confounding variables.

**Results:**

We identified three types of plant-based dietary patterns, namely, low tuber starches and vegetable plant-based diet (LTVP), high cereal, tuber starches and vegetable plant-based diet (HCTVP), and high legume and fruit plant-based diet (HLFP). We also identified three types of animal-based dietary patterns, namely, high milk and egg animal-based diet (HMiEA), low egg and fish animal-based diet, and high meat and fish animal-based diet (HMeFA). Significant coefficients for participant serum uric acid levels were observed for the HCTVP diet (*β* = −0.022, *P* = 0.031) and HMeFA diet (*β* = 0.061, *P* < 0.001). The median intake of foods in the HCTVP diet was as follows: cereals and cereal products, 444.83 g/d; tubers and starch products, 166.67 g/d; dried legumes and legume products, 8.33 g/d; vegetables and vegetable products, 333.33 g/d; and fruits and fruit products, 0 g/d. The median intake of foods in the HMeFA diet was as follows: meat and meat products, 73.33 g/d; poultry and poultry products, 0 g/d; milk and milk products, 0 g/d; eggs and egg products, 26.67 g/d; and fish, shellfish, and mollusks, 180.00 g/d.

**Conclusion:**

We showed that individual serum uric acid levels (1) might decrease under the plant-based HCTVP diet, (2) might increase under the animal-based HMeFA diet, (3) might not decrease under the plant-based HLFP diet, and (4) might not increase under the animal-based HMiEA diet. Further studies are needed to confirm these associations.

**Supplementary Information:**

The online version contains supplementary material available at 10.1186/s12937-023-00885-2.

## Background

Serum uric acid has been found to be associated with the incidence and progression of hyperuricemia, gout, chronic kidney disease, hypertension, cardiovascular events, diabetes, rheumatoid arthritis, and obesity [1–7]. Therefore, it is a priority for public health to focus on controlling individuals’ serum uric acid levels.

Understanding an individual’s diet is essential to lowering their serum uric acid levels [8]. According to previous studies, high intakes of protein, lipids and fructose and low intakes of glucose are most common in patients with hyperuricemia [9–15]. After adjustment for demographic characteristics, a positive association has been found between carbohydrate and lipid intake and serum uric acid levels [16]. Additionally, it has been reported that Vitamin D deficiency increases the risk of hyperuricemia, with a pooled odds ratio of 1.496 (95% confidence interval − 1.141, 1.963) [17]. Some previous studies have shown that plant-based foods like legumes and fruits are beneficial for the decreasing of the individuals’ serum uric acid levels, whereas animal-based foods like meats, animal giblets, and fish are harmful to the decrease of the serum uric acid level [18, 19]. The mentioned studies mainly focus on single or a few nutrients or foods. However, it is crucial to recognize that in the real world, humans do not consume isolated nutrients or foods. Therefore, relying solely on understanding the effects of singular or limited nutrients/foods on serum uric acid levels has certain limitations.

In contrast to these limited-food studies, dietary patterns have certain advantages in integrating the complex interactions of various nutrients or foods in the human body. Studies have found that the Mediterranean and Dietary Approaches to Stop Hypertension dietary patterns are beneficial in reducing serum uric acid levels among overweight or obese individuals [20]. However, to the best of our knowledge, there are only a few studies investigating the relationship of different types of plant-based or animal-based dietary patterns with individual serum uric acid levels, with limited comparisons between these dietary patterns. The objective of this study was to examine the relationship between different plant- or animal-based dietary patterns and individual serum uric acid levels. This research will help in designing a targeted dietary pattern to regulate serum uric acid levels among individual patients.

## Materials and methods

### Study population

Study participants were from the sixth wave of the China Health and Nutrition Survey (CHNS). Briefly, the CHNS collected health data from 228 communities spanning nine diverse provinces using the multistage random cluster sampling method. In the sixth wave, conducted in 2009, blood and urine samples were tested for the first time [21, 22]. We excluded participants with incomplete data (including demographic characteristics, diet, biomarkers, and lifestyle characteristics such as smoking and physical activity), as well as those aged below 18 years [23].

The survey was approved by the ethics committee of the University of North Carolina at Chapel Hill and the National Institute for Nutrition and Health, Chinese Center for Disease Control and Prevention.

### Dietary consumption assessment

Three consecutive days of 24-hour dietary recalls onsite were used to collect information regarding participants’ food consumption. Then, we identified 10 types of foods, including five types of plant-based foods and five types of animal-based foods with intakes of > 10 g per day, and calculated the nutrients in each food, according to the *Chinese Food Composition Tables*. See details in the supplementary material. For each type of food, two parts were calculated. One part was unprocessed food (such as cereals), and the other part was processed food (such as cereal products). Latent profile analysis (LPA) was used to identify different type of dietary pattern. The fit indices of LPA were log-likelihood (LogL), Akaike information criterion (AIC), Bayesian information criterion (BIC), adjusted BIC (aBIC), adjusted AIC (aAIC), entropy, Lo-Mendell-Rubin likelihood ratio test (LMRT), and the bootstrap likelihood ratio test (BLRT). See details in Tables 2 and 3.

### Serum uric acid level assessment

Fasting blood samples were taken from participants, stored at − 70 °C, and then analyzed under stringent quality control in the national central lab. The serum uric acid levels were analyzed using the enzymatic colorimetric method. Details of the biomarker analysis methods and quality control standards are presented in a previous publication (*Manual for Specimen Collection and Processing*) [24].

### Measurements of covariates

The other variables in the study were demographic characteristics including age, sex, marital status, education level, registered residence type, body mass index (BMI) and lifestyle factors including smoking and physical activity. We classified marital status into as unmarried, married, divorced, widowed, and separated. We classified education level as illiterate/primary school, middle school/high school, and above high school. We classified registered resident type into urban and rural. Body mass index (BMI) was calculated according to the equation: BMI = body weight (kg) / height^2^ (m^2^). Height was measured using the upright height measurement method, and weight was measured after removing heavy objects such as shoes and jewelry. We classified smoking status into as none, ever smoker, and current smoker. Physical activity was calculated for all activities within 1 week, and the metabolic equivalent of each task per week was calculated [25, 26]. Sedentary time was calculated for all sitting time within 1 week. We considered four types of physical activity in the study: domestic activities, occupational activities, transportation activities, and leisure activities [27].

### Statistical analysis

The variables are presented as *mean* (s*tandard deviation*), range, or median (25th percentile, 75th percentile) for continuous variables, and the proportions of the total for categorical variables. We used the *t-*test, *F* test, and *χ*^*2*^ test to determine differences in the distribution of serum uric acid levels according to demographic characteristics. The Mann–Whitney *U* test was used to determine differences in the distribution of foods and nutrient intakes according to dietary patterns. The Bonferroni test was used in post hoc multiple comparisons. We used Pearson or Spearman correlation method to analyze the correlations between variables. Linear regression analyses was used to explore the associations between dietary pattern and serum uric acid levels. Both an unadjusted model and adjusted model were applied in the analyses. The adjusted model controlled for demographic characteristics and lifestyle characteristics. Significance was based on two-sided tests, and the confidence interval was 95%.

## Results

### Demographic characteristics

A total of 7,806 participants (4,180 female, 53.54% and 3,626 male, 46.45%) aged 18–94 years with average age 50 (15) years were included in the study. Among all participants, 70.52% were from rural areas, and 88.39% had an education level of high school or below. Participants were from communities with an average urban index of 66.83 (19.40). Participants’ average BMI and physical activity range were 23.36 (3.47) kg/m^2^ and 0–31,830.00, respectively.

The correlation of participants’ serum uric acid levels with urban index and BMI were all positive, with correlation coefficients *r* = 0.092 and *r* = 0.185 (*P* < 0.001), respectively. The correlation of participants’ serum uric acid levels with the physical activity was negative, with correlation coefficient *r* = − 0.023 (*P* = 0.040). Participants who were older, male, separated, from urban areas, ever smokers, and those with higher education levels had significantly higher serum uric acid levels (*P* < 0.01). See Table 1 for details.

**Table 1:** Participants’ demographic characteristics and the distribution of serum uric acid levels (*n* = 7806)

Characteristic	*N*(*%*)	Serum uric acid level (mg/mL)		
		*Mean* (*SD*)	*t*/*F*	*P*
Age 18–59 years 60- years	5627 (72.08) 2179 (27.91)	5.09 (1.86) 5.39 (4.56)	7.131	<0.001
Gender Female Male	4180 (53.54) 3626 (46.45)	4.48 (1.36) 5.96 (1.89)	39.152	<0.001
Marital status Unmarried Married Divorced Widowed Separated	445 (5.70) 6625 (84.87) 79 (1.01) 609 (7.80) 48 (0.61)	5.46 (2.31) 5.15 (1.77) 5.00 (1.48) 5.18 (1.52) 5.64 (1.57)	4.202	0.002
Education level Illiterate/primary Middle school/high school Above high school	3443 (44.11) 3457 (44.29) 906 (11.61)	5.06 (1.67) 5.18 (1.75) 5.58 (2.27)	12.000	<0.001
Registered resident type Urban Rural	2301 (29.48) 5505 (70.52)	5.39 (1.94) 5.08 (1.71)	6.572	<0.001
Smoking None Ever Current	5400 (69.18) 255 (3.27) 2151 (27.56)	4.88 (1.70) 5.91 (1.62) 5.83 (1.81)	257.677	<0.001

### Latent profiles of dietary patterns

We examined latent profiles of participants’ plant-based dietary patterns. The LPA model fitting parameters are listed in Table 2. Model fit information for the five different models is listed, ranging from Profile 2 to Profile 5. In terms of the LMRT and BLRT, *P*-values for the Profile 2 and Profile 3 models were both < 0.05 (statistically significant). Profile 3 had lower AIC and BIC values compared with Profile 2. The entropy in Profile 3 was 0.937 > 0.800. We found that the accuracy of classification was greater than 90.00% [29]. The Profile 3 model was better than the other models in this study.

**Table 2:** Fit indices for Profile 2 through 5 models

*Profiles*	*LogL*	*AIC*	*BIC*	*aBIC*	*Entropy*	*LMRT*	*BLRT*
2	-236220.541	472473.082	472584.485	472533.640	0.954	0.018	< 0.01
3	-234906.914	469857.828	470011.006	469941.095	0.935	< 0.001	< 0.001
4	-233765.886	467587.773	467782.727	467693.749	0.937	0.435	< 0.001
5	-232925.060	465918.121	466154.851	466046.806	0.933	0.115	< 0.001

The latent profiles of participants’ animal-based dietary patterns were examined. The LPA model fitting parameters are listed in Table 3. The model fit information for the five different models is listed, ranging from Profile 2 to Profile 5. In terms of the LMRT and BLRT, *P*-values for the Profile 2 and Profile 3 models were both < 0.05 (statistically significant). Profile 3 had lower AIC and BIC values compared with Profile 2. The entropy in Profile 3 was 0.968 > 0.800. The accuracy of classification was greater than 90.00% [28]. The Profile 3 model was better than the other models in this study.

**Table 3:** Fit indices for Profile 2 through 5 models

*Profiles*	*LogL*	*AIC*	*BIC*	*aBIC*	*Entropy*	*LMRT*	*BLRT*
2	-201762.416	403556.832	403668.234	403617.389	0.999	0.321	< 0.01
3	-200530.816	401105.632	401258.811	401188.899	0.968	0.047	< 0.001
4	-199170.235	398396.470	398591.424	398502.445	0.966	0.483	< 0.001
5	-198071.225	396210.449	396447.179	396339.134	0.957	0.426	< 0.001

### Food and nutrient intake in different latent profiles of dietary patterns

The food and nutrient intakes for each identified profile of plant-based dietary patterns are shown in Tables 4 and 5.

We found that participants with a Profile 2 dietary pattern had higher intake of cereals and cereal products, tuber starches and products, and vegetables and vegetable products than those with Profiles 1 and 3 (*P* < 0.001). Participants with a Profile 3 dietary pattern had higher intakes of dried legumes and legume products, and fruit and fruit products than those with Profiles 1 and 2 (*P* < 0.001). Participants with a Profile 1 dietary pattern had lower intakes of tuber starches and products, and vegetables and vegetable products than those with Profiles 2 and 3 (*P* < 0.001). See Table 4 for details.

The characteristics of the Profile 1 dietary pattern comprised the lowest intakes of tuber starches and products and of vegetables and vegetable products. The characteristics of the dietary pattern in Profile 2 showed higher intakes of cereals and cereal products, tubes starches and products, and vegetables and vegetable products. The characteristics of the dietary patterns in Profile 3 included the highest intake of dried legumes and legume products and fruit and fruit products. Based on the characteristics of these plant-based dietary pattern profiles, we denoted Profile 1–3 dietary patterns as the low tuber starches and vegetable plant-based diet (LTVP), high cereal, tuber starches, and vegetable plant-based diet (HCTVP), and high legume and fruit plant-based diet (HLFP), respectively. See Table 4 for details.

Participants with a LTVP dietary pattern had the highest intakes of vitamin A and calcium (*P* < 0.01). We also found that participants with an HCTVP dietary pattern had the highest intake of energy, lipids, carbohydrate, protein, dietary fiber, thiamine, riboflavin, niacin, vitamin C, vitamin E, phosphorus, potassium, sodium, magnesium, iron, zinc, selenium, copper, and manganese (*P* < 0.01). Participants with an HCTVP dietary pattern had higher intakes of protein, lipids, thiamine, riboflavin, niacin, vitamin C, vitamin E, phosphorus, potassium, sodium, iron, zinc, selenium, copper, and manganese than participants with an LTVP dietary pattern (*P* < 0.01). See Table 5 for details.

In the study, the proportion of participants who reported having LTVP, HCTVP, and HLFP was 85.0%, 8.3%, and 6.6%, respectively. There were correlations between animal-based dietary pattern and sex (*χ*^*2*^ = 24.690, *P* < 0.001), education level (*χ*^*2*^ = 217.866, *P* < 0.001), age (*F* = 5.631, *P* = 0.004), and urban index (*F* = 146.107, *P* < 0.001).

**Table 4:** Daily dietary food intakes in latent profiles of plant-based dietary patterns

	*All*			*Latent Dietary Patterns* (*P*_*50*_)		
Food intakes	*P*_*25*_	*P*_*50*_	*P*_*75*_	*Pattern 1* *LTVP*	*Pattern 2* *HCTVP*	*Pattern 3* *HLFP*
Cereals and cereal products (g)	283.330	366.670	480.000	363.330	444.830	318.335
Tubers starches and products (g)	0	0	56.670	0	166.670	33.330
Dried legumes and legume products (g)	0	33.330	83.330	33.330	8.330	50.000
Vegetables and vegetable products (g)	200.000	300.000	408.330	300.000	333.330	306.670
Fruit and fruit products (g)	0	0	66.670	0	0	330.000

**Table 5:** Daily dietary nutrient intakes in latent profiles of plant-based dietary patterns

	*All*			*Latent Dietary Patterns* (*P*_*50*_)		
Nutrients intake	*P*_*25*_	*P*_*50*_	*P*_*75*_	*LTVP*	*HCTVP*	*HLFP*
Energy (kcal)	2953.00	3571.00	4302.50	3556.00	3575.00	3769.50
Protein (g)	84.20	107.30	139.20	108.20	100.40	109.75
Lipid (g)	12.00	21.10	40.00	21.80	14.90	26.00
Carbohydrate (g)	612.10	755.40	890.90	752.00	772.80	794.80
Dietary fiber (g)	19.90	27.40	39.70	27.10	26.00	33.15
Cholesterol (mg)	0.00	0.00	0.00	0.00	0.00	0.00
Vitamin A (mg)	295.00	644.00	1201.00	684.00	384.00	638.00
Thiamine (mg)	1.34	1.73	2.30	1.71	1.77	1.90
Riboflavin (mg)	0.96	1.19	1.52	1.19	1.13	1.34
Niacin (mg)	19.50	23.80	28.91	23.50	24.92	25.38
Vitamin C (mg)	151.00	226.00	334.00	221.00	243.00	269.50
Vitamin E (mg)	14.67	23.05	38.86	22.87	19.85	32.29
Calcium (mg)	538.00	757.00	1047.00	772.00	597.00	771.50
Phosphorus (mg)	1421.00	1787.00	2345.00	1792.00	1667.00	1832.00
Potassium (mg)	2664.75	3449.00	4418.25	3368.00	3756.00	4141.00
Sodium (mg)	365.48	637.05	1083.30	647.00	544.00	661.05
Magnesium (mg)	494.75	630.00	844.00	625.00	620.00	698.50
Iron (mg)	31.60	39.90	52.60	40.10	37.20	40.90
Zinc (mg)	15.98	19.50	24.14	19.53	18.55	20.47
Selenium (mg)	36.92	52.68	71.72	53.00	50.60	51.49
Copper (mg)	3.23	4.17	5.45	4.13	4.04	4.93
Manganese (mg)	11.43	14.32	18.03	14.41	13.26	14.54

The intakes of foods and nutrients in each identified profile of animal-based dietary patterns are shown in Tables 6 and 7.

Participants with a Profile 1 dietary pattern had higher intakes of milk and milk products and eggs and egg products than those with Profile 2 and 3 dietary patterns (*P* < 0.001). Participants with a Profile 3 dietary pattern had higher intakes of meat and meat products and fish shellfish, and mollusks. Participants with a Profile 2 dietary pattern had lower intakes of eggs and egg products, fish shellfish, and mollusks than those with Profiles 1 and 3 (*P* < 0.001). See Table 6 for details.

The characteristics of the Profile 1 dietary pattern included highest intakes of milk and milk products and eggs and egg products. The characteristics of the dietary patterns in Profile 2 included the lowest intakes of eggs and egg products and fish, shellfish, and mollusks. The characteristics of the dietary patterns in Profile 3 included the highest intakes of meat and meat products and fish, shellfish, and mollusks. Based on the characteristics of these animal-based dietary pattern profiles, we denoted the Profile 1–3 dietary patterns as the high milk and egg animal-based diet (HMiEA), low egg and fish animal-based diet (LEFA), and high meat and fish animal-based diet (HMeFA), respectively. See Table 6 for details.

Participants with an HMiEA dietary pattern had the highest intakes of energy, carbohydrate, cholesterol, vitamin A, thiamine, riboflavin, vitamin C, calcium, phosphorus, potassium, sodium, and selenium (*P* < 0.01). Participants with an HMeFA dietary pattern had the highest intakes of protein, lipids, niacin, vitamin E, magnesium, iron, zinc, copper, and manganese (*P* < 0.01). See Table 7 for details.

In the study, the proportion of participants who reported having LTVP, HCTVP, and HLFP was 4.6%, 89.8%, and 5.6%, respectively. There were correlations between an animal-based dietary pattern and sex (*χ*^*2*^ = 25.038, *P* < 0.001), education level (*χ*^*2*^ = 269.214, *P* < 0.001), age (*F* = 11.564, *P* < 0.001), and urban index (*F* = 168.029, *P* < 0.001).

**Table 6:** Daily dietary food intakes in latent profiles of animal-based dietary patterns

	*All*			*Latent Dietary Patterns* (*P*_*50*_)		
Food intakes	*P*_*25*_	*P*_*50*_	*P*_*75*_	*Pattern 1* *HMiEA*	*Pattern 2* *LEFA*	*Pattern 3* *HMeFA*
Meat and meat products (g)	16.670	60.000	108.330	66.670	60.000	73.330
Poultry and poultry products (g)	0	0	0	0	0	0
Milk and milk products (g)	0	0	0	222.000	0	0
Eggs and egg products (g)	0	20.000	48.330	40.000	20.000	26.670
Fish shellfish and mollusc (g)	0	0	50.000	28.330	0	180.000

**Table 7:** Daily dietary nutrient intakes in latent profiles of animal-based dietary patterns

	*All*			*Latent Dietary Patterns* (*P*_*50*_)		
Nutrients intake	*P*_*25*_	*P*_*50*_	*P*_*75*_	*HMiEA*	*LEFA*	*HMeFA*
Energy (kcal)	600.00	1212.00	1940.00	1757.00	1150.00	1734.00
Protein (g)	39.90	78.85	120.83	122.10	71.20	133.70
Lipid (g)	39.50	91.60	154.83	120.60	86.85	124.40
Carbohydrate (g)	5.60	11.10	18.60	28.10	10.20	18.80
Dietary fiber (g)	0.00	0.00	0.00	0.00	0.00	0.00
Cholesterol (mg)	505.00	1065.00	1755.00	1626.00	985.00	1608.00
Vitamin A (mg)	126.00	346.50	628.00	648.00	324.00	532.00
Thiamine (mg)	0.38	0.78	1.36	1.21	0.74	1.02
Riboflavin (mg)	0.52	0.91	1.40	1.65	0.85	1.38
Niacin (mg)	6.00	14.00	23.70	20.05	12.65	22.10
Vitamin C (mg)	0.00	0.00	0.00	2.00	0.00	0.00
Vitamin E (mg)	2.19	4.40	7.25	6.96	4.04	9.10
Calcium (mg)	62.00	137.00	259.00	516.00	121.00	385.00
Phosphorus (mg)	422.00	810.00	1248.50	1414.00	746.00	1398.00
Potassium (mg)	559.00	1060.00	1702.25	1961.00	970.00	1934.00
Sodium (mg)	244.60	441.20	789.00	875.50	400.40	813.80
Magnesium (mg)	42.00	89.00	148.00	161.00	80.00	188.00
Iron (mg)	5.00	9.40	15.40	14.90	8.80	15.60
Zinc (mg)	4.26	8.97	14.68	14.05	8.24	14.11
Selenium (mg)	35.91	66.90	105.51	108.69	61.50	130.75
Copper (mg)	0.27	0.50	0.88	0.87	0.46	0.97
Manganese (mg)	0.10	0.20	0.36	0.37	0.18	0.51

### Relationship between individual serum uric acid levels and dietary patterns

There was no significant difference in participants’ serum uric acid levels according to different types of plant-based dietary patterns (*F* = 1.176, *P >* 0.05). We found a significant difference in serum uric acid levels between different types of animal-based dietary patterns (*F* = 32.792, *P <* 0.001). Participants who followed an HMeFA diet (participants’ mean serum uric acid 5.82 mg/mL) had higher serum uric acid levels than those who had an HMiEA diet (participants’ mean serum uric acid 5.12) and LEFA diet (participants’ mean serum uric acid 5.12) (*P <* 0.01).

Table 8 shows the association between the plant/animal-based dietary pattern and participants’ serum uric acid levels. In the unadjusted model, significant coefficients for serum uric acid levels were observed for the HEMA diet (*β* = 0.027, *P* = 0.018) and the HMeFA diet (*β* = 0.089, *P* < 0.001). Furthermore, in the adjusted model, significant coefficients for participants’ serum uric acid levels were observed for the HCTVP diet (*β* = −0.022, *P* = 0.031) and HMeFA diet (*β* = 0.061, *P* < 0.001).

**Table 8:** Association between dietary patterns and serum uric acid levels

		Serum uric acid level			
		Model I†		Model II‡	
		*β*	*P*	*β*	*P*
Plant-based dietary pattern	*LTVP*	0.017	0.252		
	*HCTVP*			-0.022	0.031
	*HLFP*	-0.003	0.823	<0.001	0.998
Animal-based dietary pattern	*HMiEA*	0.027	0.018	0.016	0.11
	*LEFA*				
	*HMeFA*	0.089	<0.001	0.061	<0.001

† Model I was the unadjusted model. For plant-based dietary patterns, the HCTVP pattern was the reference. For the plant-based dietary patterns, LEFA was the reference

‡ Model II was adjusted for sex, age, marital status, education level, registered resident type, urban index, BMI, smoking and physical activity. For plant-based dietary patterns, the LTVP pattern was the reference. For the plant-based dietary patterns, LEFA was the reference

## Discussion

In the present cross-sectional study, we assessed the effect of different plant/animal-based dietary pattern on individual serum uric acid levels in Chinese adults. We found one harmful and one beneficial dietary pattern to decrease serum uric acid levels.

Among the three plant-based dietary patterns, only the HCTVP diet might be beneficial for decreasing serum uric acid levels. The HCTVP diet (cereals, cereal products, tuber starches and products) is characterized by high intakes of carbohydrate and starch and lower intakes of protein and lipids. Our findings are consistent with those of previous studies. For instance, Johnston et al. investigated 20 healthy adults and reported that after 6 weeks’ consumption of a high carbohydrate diet, they observed a 22–30% decrease in individuals’ serum uric acid levels [29]. Another study among 12,765 adults in Australian and Tromso reported that higher consumption of carbohydrate and lower consumption of lipids were associated with decreased individual serum uric acid levels [30]. One possible reason for this finding might be that high carbohydrate intake could slow down the decrease in gluconeogenesis disorders, which decreases the pentose phosphate pathway and the serum uric acid level [31]. Another reason may be that high carbohydrate foods like cereals contain high levels of glucose, which could increase the excretion of uric acid in the kidney and decrease the uric acid in serum [17]. The HCTVP diet (vegetables) was also characterized by a low intake of fructose foods. Previous studies have reported that fructose consumption is a risk factor of increased serum uric acid levels [32]. There are two possible mechanisms underlying this effect of fructose on serum uric acid. One is that fructose consumption would lead to the reactant aggregation of uric acid, and finally lead to increasing serum uric acid levels [33, 34]. The other mechanism is that fructose consumption might result in insulin resistance, which would increase gluconeogenesis disorders and the pentose phosphate pathway, finally leading to an increase in serum uric acid levels [31, 35, 36].

Thus, our findings suggested that only the plant-based HCTVP diet that comprised high intakes of cereals, tuber starches, and vegetables might have a beneficial impact on decreasing the individuals’ serum uric acid levels.

Of the three animal-based dietary patterns, only the HmeFA diet might be leading to increasing the levels of individual serum uric acid. The HmeFA diet was characterized as having a high intake of purine foods (meats, meats products, fish, shellfish, and mollusks). Our findings are consistent with those of previous studies [37]. For instance, Choi et al. studied 14,809 adults over 20 years old and reported that higher levels of meat and seafood consumption were associated with higher serum levels of uric acid [38]. The reason is that uric acid is a product of purine metabolism in the body. If individuals consume a high-purine diet, their serum uric acid would be at a high level for a long time, which might cause damage to renal function, leading to a further rise in serum uric level. The HmeFA diet was characterized as having a high intake of lipids. The mechanism underlying the lipid-related increase in uric acid is that high intakes of fatty acids can cause oxidative stress in the body and impair kidney function, which might cause serum uric levels to rise further [39]. Another characteristic of the HmeFA diet was a high intake of niacin. Our data are similar to those of previous studies. For instance, Kei and ElisafIt indicated that niacin treatment would lead to a side effect of hyperuricemia (incidence rate 14%) [40]. Another study showed that niacin treatment would decrease uric acid renal clearance, followed by an increase in serum uric acid level [41]. A possible mechanism underlying the effect of niacin on individual serum uric acid levels is that niacin would lead to hepatic insulin resistance and finally lead to increasing serum uric acid [42].

Thus, our findings suggested that only the animal-based HmeFA diet with high meat and fish consumption might have a harmful impact on decreasing individual serum uric acid levels.

There are several limitations in this study. First, the dietary data in this study were obtained from three consecutive 24-hour dietary recalls, which could yield measurement error versus non-consecutive and long-term recalls. Additionally, we used cross-sectional data; therefore, causal inference cannot be made.

## Conclusion

We found a difference between different types of plant-based and animal-based dietary patterns in terms of effect on individual serum uric acid levels. Only the plant-based dietary pattern HCTVP and animal-based dietary pattern HmeFA influenced serum uric acid levels. Therefore, to reduce high levels of serum uric acid, following the HCTVP diet rather than the HLFP or LTVP dietary pattern is recommended; the HMeFA diet should be avoided. Future studies should be conducted to verify the proper amounts of nutrients and composition in specially designed dietary patterns.

### Electronic supplementary material

Below is the link to the electronic supplementary material.

Supplementary Material 1

## Data Availability

The complete data can be found at https://www.cpc.unc.edu/projects/china.
