# Supplementary material for: Analysis of different plant- and animal-based dietary patterns and their relationship with serum uric acid levels in Chinese adults
Source: Nutr J. 2023 Oct 27;22:53. doi: 10.1186/s12937-023-00885-2 (PMC10605343; doi:10.1186/s12937-023-00885-2)
Supplement: Supplementary file 1 — Supplementary Material 1 [file 12937_2023_885_MOESM1_ESM.docx]

**Table 9 Daily dietary food intakes of 21 types of foods in the the Chinese Food Composition Tables (*n*=7806)**

| Food intakes (g) | *P_25_* | *P_50_* | *P_75_* | *Mean* | *Standard Deviation* |
| --- | --- | --- | --- | --- | --- |
| Cereals and cereal products | 283.33 | 366.67 | 480.00 | 392.75 | 171.81 |
| Vegetables and vegetable products | 200.00 | 300.00 | 408.33 | 322.20 | 171.17 |
| Meat and meat products | 16.67 | 60.00 | 108.33 | 73.20 | 69.94 |
| Dried legumes and legume products | 0.00 | 33.33 | 83.33 | 55.67 | 75.69 |
| Fruit and fruit products | 0.00 | 0.00 | 66.67 | 55.38 | 110.49 |
| Tubers starches and products | 0.00 | 0.00 | 56.67 | 36.21 | 59.10 |
| Fish shellfish and mollusc | 0.00 | 0.00 | 50.00 | 33.98 | 58.71 |
| Eggs and egg products | 0.00 | 20.00 | 48.33 | 30.47 | 36.95 |
| Poultry and poultry products | 0.00 | 0.00 | 0.00 | 15.22 | 35.64 |
| Milk and milk products | 0.00 | 0.00 | 0.00 | 12.43 | 50.45 |
| Ethnic foods and cakes | 0.00 | 0.00 | 0.00 | 9.20 | 35.09 |
| Fast foods | 0.00 | 0.00 | 0.00 | 9.16 | 26.51 |
| Liquor and alcoholic beverages | 0.00 | 0.00 | 0.00 | 8.74 | 62.44 |
| Condiments | 0.00 | 0.00 | 3.00 | 6.76 | 17.84 |
| Fungi and algae | 0.00 | 0.00 | 0.00 | 5.59 | 18.95 |
| Beverages | 0.00 | 0.00 | 0.00 | 4.45 | 47.64 |
| Nuts and seeds | 0.00 | 0.00 | 0.00 | 4.15 | 20.03 |
| Others | 0.00 | 0.00 | 0.00 | 0.20 | 3.34 |
| Sugars and preserves | 0.00 | 0.00 | 0.00 | 0.18 | 2.97 |
| Infant foods | 0.00 | 0.00 | 0.00 | 0.05 | 1.20 |
| Fats and oils | 0.00 | 0.00 | 0.00 | 0.03 | 1.36 |

**Table 10 Participants’ continuous demographic characteristics and the distribution of serum uric acid levels (*n*=7806)**

| Characteristic | *Mean* (*SD*) | Range | Serum uric acid level (mg/mL) | |
| --- | --- | --- | --- | --- |
|  |  |  | *r* | *P* |
| Urban index | 66.83 (19.40) | 30.42-106.50 | 0.092 | ＜0.001 |
| BMI | 23.36 (3.47) | 13.70-42.72 | 0.158 | ＜0.001 |
| Physical activity | 1142.21(3556.36) | 0–31,830.00 | -0.014 | 0.231 |
